# Contextual facilitation: Separable roles of contextual guidance and context suppression in visual search

**DOI:** 10.3758/s13423-024-02508-1

**Published:** 2024-04-30

**Authors:** Siyi Chen, Hermann J. Müller, Zhuanghua Shi

**Affiliations:** Allgemeine und Experimentelle Psychologie, Department Psychologie, LMU München, Leopoldstr. 13, D-80802 Munich, Germany

**Keywords:** Contextual cueing, Visual search, Context-based guidance, Context suppression

## Abstract

Visual search is facilitated when targets are repeatedly encountered at a fixed position relative to an invariant distractor layout, compared to random distractor arrangements. However, standard investigations of this *contextual-facilitation* effect employ fixed distractor layouts that predict a constant target location, which does not always reflect real-world situations where the target location may vary relative to an invariant distractor arrangement. To explore the mechanisms involved in contextual learning, we employed a training-test procedure, introducing not only the standard full-repeated displays with fixed target-distractor locations but also distractor-repeated displays in which the distractor arrangement remained unchanged but the target locations varied. During the training phase, participants encountered three types of display: full-repeated, distractor-repeated, and random arrangements. The results revealed full-repeated displays to engender larger performance gains than distractor-repeated displays, relative to the random-display baseline. In the test phase, the gains were substantially reduced when full-repeated displays changed into distractor-repeated displays, while the transition from distractor-repeated to full-repeated displays failed to yield additional gains. We take this pattern to indicate that contextual learning can improve performance with both predictive and non-predictive (repeated) contexts, employing distinct mechanisms: contextual guidance and context suppression, respectively. We consider how these mechanisms might be implemented (neuro-)computationally.

## Introduction

Goal-related targets in our everyday life do not appear in isolation, they coexist with multiple non-target items in a scene. When the target’s location remains stable relative to its surroundings, our ability to find it improves, owing to the learning of the invariant spatial relations – known as “contextual cueing” (e.g., Chun & Jiang, 1998; Geyer et al., 2010). In a typical contextual-cueing study (Chun & Jiang, 1998), observers search for a target “T” amongst distractor “Ls” – a task that offers minimal feature-based search guidance (Moran et al., 2016). Critically, unbeknown to observers, the spatial arrangements of the target and distractors are repeated in half of the trials (“old” contexts) and random in the other half (“new” contexts). One common finding is that just a few repetitions of “old” contexts, interspersed among random-context displays, suffice to facilitate search performance, often without explicit recognition of “old” contexts (but see Annac et al., 2019; Vadillo et al., 2016).

Since Chun and Jiang’s (1998) seminal study, most investigations of contextual cueing have adopted similar experimental setups, randomly repeating exact old contexts multiple times throughout the experiment, without altering their configural predictiveness (Chun, 2000; Chun & Jiang, 1999; Geyer et al., 2010; Goujon et al., 2015; Shi et al., 2013; Sisk et al., 2019; Wolfe & Horowitz, 2017). The results support the attention-guidance account (Chun & Jiang, 1998), according to which learnt spatial associations between the target and distractors (stored in long-term memory and activated by a given old display) “cue” attention to – or predict – the target location (e.g., Brockmole & Henderson, 2006; Chen et al., 2021; Geyer et al., 2010; Giesbrecht et al., 2013; Johnson et al., 2007; Peterson & Kramer, 2001; Schlagbauer et al., 2017; Tseng & Li, 2004; Zinchenko et al., 2020). Additionally, contextual cueing might also be attributable (in part) to facilitation of the late processing stage of response selection and/or motor execution, when participants decide which motor (hand) effector to use for a correct response (e.g., Chen et al., 2021; Hout & Goldinger, 2012; Kunar et al., 2007; Schankin & Schubö, 2010).

However, learnt predictive spatial contexts may not always be beneficial. When the target changes its position while the non-target items remain at their locations, learnt contextual layouts can impede search performance – because attention is misguided to the old target location (Manginelli & Pollmann, 2009; Zellin et al., 2013; Zinchenko et al., 2020). Interestingly, when the fixed distractor layout does not predict the target location during learning (e.g., when the target changes its location on every trial, while the non-target items stay in their old positions), such non-predictive “old” contexts can still facilitate search (Vadillo et al., 2021; Wang et al., 2020). Vadillo and colleagues (2021) argued that, in this case, search is facilitated by inhibiting the locations occupied by the old distractors, making searching more efficient by (relative to the distractors) increasing the attentional priority of the target. These findings align with a core assumption of Beesley et al.’s (2015) connectionist model scheme, namely, that contextual cueing may involve not only learning to predict the target location, but also acquiring associations among the distractor locations themselves. That is, either the target-distractor relations or the distractor-context alone can be effectively learned.^1^

In more “cognitive” terms, the contextual-*guidance* (e.g., Manginelli & Pollmann, 2009; Zellin et al., 2013; Zinchenko et al., 2020) and the context-*suppression* accounts (Vadillo et al., 2021) may be understood to assume a qualitative difference in the learnt search modes. Observers may come to adopt either a contextual-guidance mode for full-repeated displays or a context-suppression mode for distractor-repeated displays – depending on the non-/predictability of the target location from the distractor context. If observers adopt the contextual-guidance mode, transitioning from full-repeated displays to distractor-repeated displays may lead to performance costs, due to the persistent miscueing of attention to the original target location, which takes extended training to unlearn (e.g., Manginelli & Pollmann, 2009; Zellin et al., 2013; Zinchenko et al., 2020). In contrast, there may be little cost when transitioning from distractor-repeated to full-repeated displays: the suppression strategy does not depend on the target’s location, and so the learnt distractor context can still be effectively suppressed, while opening the possibility of acquiring contextual-guidance “cues,” and so affording operation in the “guidance” search mode.

The aim of the present study was to investigate the interplay of the contextual-guidance and -suppression search modes when the predictivity, or, respectively, non-predictivity, of the distractor context with respect to the target location changes from an initial learning to a subsequent test phase. During the training phase, one-third of displays consisted of full repetitions of the (fixed) target-context arrangements (full-repeated displays). In another third, the arrangement of the distractors remained identical across repetitions, as in full-repeated displays, but the location of the target changed across the repetitions (distractor-repeated displays). The remaining third comprised displays with all distractors appearing at random locations (new, baseline displays). In the test phase, while the new displays were newly generated, the old contexts in initially full-repeated displays (from the training phase) were rendered non-predictive with respect to the target location, and the old contexts in initially distractor-repeated displays were rendered fully predictive of the target location, without altering the distractor configuration. Importantly, the number of possible target locations in each display condition was fixed (including a change of the target locations from the training to the test phase in each condition) – a standard procedure to control for influences of absolute (“context-less”) target-location learning (cf. Chun & Jiang, 1998).

### Method

#### Participants

Twenty-five participants (12 females; age: *M* = 23.25 years) took part in the study. All were right-handed and had normal or corrected-to-normal visual acuity. The sample size was determined based on a meta-analysis of the contextual-cueing effect (*d*_*z*_ = 0.60) from studies with distractor-repeated context and random target locations (Vadillo et al., 2021), which was found to be significantly smaller than the standard contextual-cueing effect (*d*_*z*_ = 0.97). We aimed for 85% power with an alpha level of .05, which yielded 22 participants. The study was approved by the Ethics Committee of the Department of Psychology, Ludwig-Maximilians-Universität, Munich. All participants provided written informed consent prior to the experiment and received 9 €/h for their service.

#### Apparatus and stimuli

The experiment was conducted in a dimly lit cabin (0.35 cd/m^2^). Participants were seated comfortably, with their heads supported by a chin rest and forehead support, and viewed the stimuli presented on a 19-in. CRT color monitor from a distance of 60 cm (CRT screen resolution: 1,024 × 768 pixels; refresh rate: 85 Hz). Event scheduling and response recording were controlled by the customized Matlab code with the Psychophysics Toolbox (Brainard, 1997; Pelli, 1997).

**Fig. 1:**
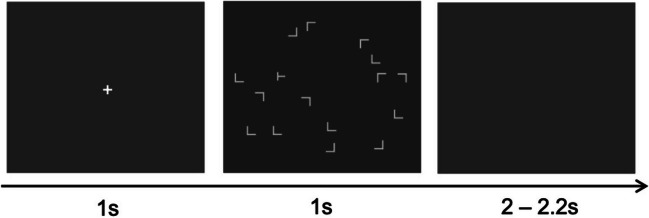
The trial sequence in the experimental paradigm. Each trial began with a central fixation marker, followed by a 1-s search display, and replaced by a blank screen for 2–2.2 s. Participants were required to respond within 2 s after display onset; otherwise, the response was invalid. The task was to find the target T among the L-type distractors and discriminate its (left/right) orientation, as quickly and accurately as possible. In the example shown, the target T is titled to the right

The search displays consisted of 16 gray items (25.38 cd/m^2^; Fig. 1), 15 of them being L-shapes, randomly rotated in one of four possible directions (0°, 90°, 180°, or 270°), and one T-shaped target, rotated either 90° or 270° (pointing to the left or right), all subtending 1.0° of visual angle. They were randomly placed at 16 of 64 possible locations, arranged around four concentric (invisible) circles with radii of 2°, 4°, 6°, and 8°, respectively, on a dark background (1.76 cd/m^2^). To avoid salient clusters, each display quadrant contained an equal number of items. The target item could only appear on the second or third ring (32 possible locations), avoiding influences of eccentricity on response speed (Zang et al., 2020).

#### Procedure and design

Each trial began with a central fixation marker (0.4°× 0.4°) for 1 s, which was followed by a 1-s search display. The search display disappeared automatically, being replaced by a blank screen. Participants had to make a response within 2 s from the onset of the search display; otherwise, the response was considered invalid. After a blank interval of 2–2.2 s, the next trial began. Participants underwent 24 practice trials, followed by 720 trials of the experiment proper. The trials were divided into 60 blocks, each of 12 trials. The first 30 blocks constituted the training phase, the subsequent 30 blocks the test phase. Participants were allowed a break after each block, with a mandatory short break every 10 blocks.

In the training phase, there were four distinct full-repeated displays, in which all elements, including the target, remained at the same location across repetitions. Each target occupied a distinct (invariant) location in one particular quadrant relative to the repeated distractor context in the respective display, with the targets appearing in different quadrants in the four displays. There were also four distinct distractor-repeated displays. In these displays, the configuration of distractors remained identical across repetitions, as in the full-repeated displays, but the target changed its location from one repetition to the next, appearing with equal probability in the four quadrants. All eight full-repeated and distractor-repeated displays were tested once within each block. The remaining four trials within each block constituted the new condition, in which all distractors appeared at random locations that differed across trials, while the target appeared equally at a position in each of the four quadrants. Thus, the target locations themselves were repeated equally in full-repeated, distractor-repeated, and new displays. That is, in each block of 12 trials, four distinct positions were used for targets in the full-repeated condition, and four positions for targets in the distractor-repeated condition (with the target positions in the full-repeated and distractor-repeated conditions never overlapping with any of the distractor locations in the two conditions), and the other four positions were used for the new condition. This was meant to ensure that any performance gains in the “repeated” conditions were attributable only to the effects of repeated spatial context-target arrangements or, respectively, the repeated contexts alone, rather than repeated target locations as such (see, e.g., Chun & Jiang, 1998, for a similar approach to rule out an influence of absolute target-location learning, cf. Geng & Behrmann, 2005).

During the test phase, the old contexts in initially full-repeated displays (from the training phase) were rendered non-predictive of the target location, by now randomizing selecting the four target locations (relative to the repeated contexts) across blocks, whereas the old contexts in initially distractor-repeated displays were rendered fully predictive of the (now fixed) target location, without altering the distractor configuration. Of note, 12 new target locations were used in the test phase, four positions for targets in the full-repeated condition, four for targets in the distractor-repeated condition, and the other four for the new condition. We selected four new, different target locations per display type in the test phase (which had not been used in the preceding training phase) to rule out that, e.g., a given target location in one type of display in the training phase was paired with the target position in another type of display in the test phase. In adopting this procedure, we effectively equated the opportunity to learn the absolute target locations (i.e., absolute target-location probability learning along the lines of Geng & Behrmann, 2005) across all three types of display, in both phases. In all other respects, the experiment remained the same as in the training phase.

After completing the formal visual search task, participants performed a “display-recognition” test, which involved 32 displays. Eight displays with old contexts (eight full-repeated displays from the training and test phases) were repeated twice, randomly interspersed with 16 newly generated displays. Participants had to classify each display as an “old” or a “new” one, pressing the left or the right arrow key, respectively.

#### Data analyses

Trials with response errors (1.6%) and those with extreme reaction times (RTs), either below 200 ms or above 2.5 standard deviations from an individual’s average RT (2.4%), were excluded from RT analysis. To achieve a reasonably stable estimate of the contextual-facilitation effect, we averaged the data over two consecutive blocks, forming one “Epoch.” Since linear-mixed-model (LMM) analysis offers a more nuanced view than standard analyses of variance (ANOVAs), in particular for the trends, here we adopted LMMs for the dependent variables (RT and accuracy), incorporating the treatment factors Epoch, Display, and Phase, as fixed effects and participant/s as the random effect. We examined whether different display types would show differential learning effects over epochs and any sudden changes between the training and test phases.

We observed a learning effect characterized by a marked decrease in RTs with increasing “epoch” of task performance, with RTs exhibiting an approximately linear relationship to the logarithmic transformation of Epoch. Performance accuracy showed less distinct trends, with accuracy fluctuating between 96% and 100%. Given this, we employed the following LMM (R formula) for the RTs:

$$RT\sim Display+log(Epoch)+log(Epoch):Display+log(Epoch):Display:Phase+(1|sub),$$

where “:” stands for an interaction and “(1|sub)” for the random effect of participant/s, and Phase is a dummy variable with 0 for the training phase and 1 for the test phase.

And we fitted the accuracy data using the following LMM:

$$Accuracy\sim Display + Epoch + Epoch:Display+Display:Phase+(1|sub).$$

We then compared the intercept estimates (*a*), reflecting RT and, respectively, accuracy in Epoch 1, and the slope estimates (*b*), indexing the changes in (RT and accuracy) performance across epochs (i.e., learning rate), among the various conditions. Finally, we tested the interaction between log(Epoch), Display, and Phase in the RT data, reflecting the change of the learning rate for each display condition across the two phases. For accuracy, we included only the interaction between Display and Phase, assuming that at most the general accuracy level would differ between the two phases. As confirmed by the AIC values associated with the above models, these models turned out to have the lowest AIC values while also being simpler compared to other models.

### Results

#### Response times

**Fig. 2:**
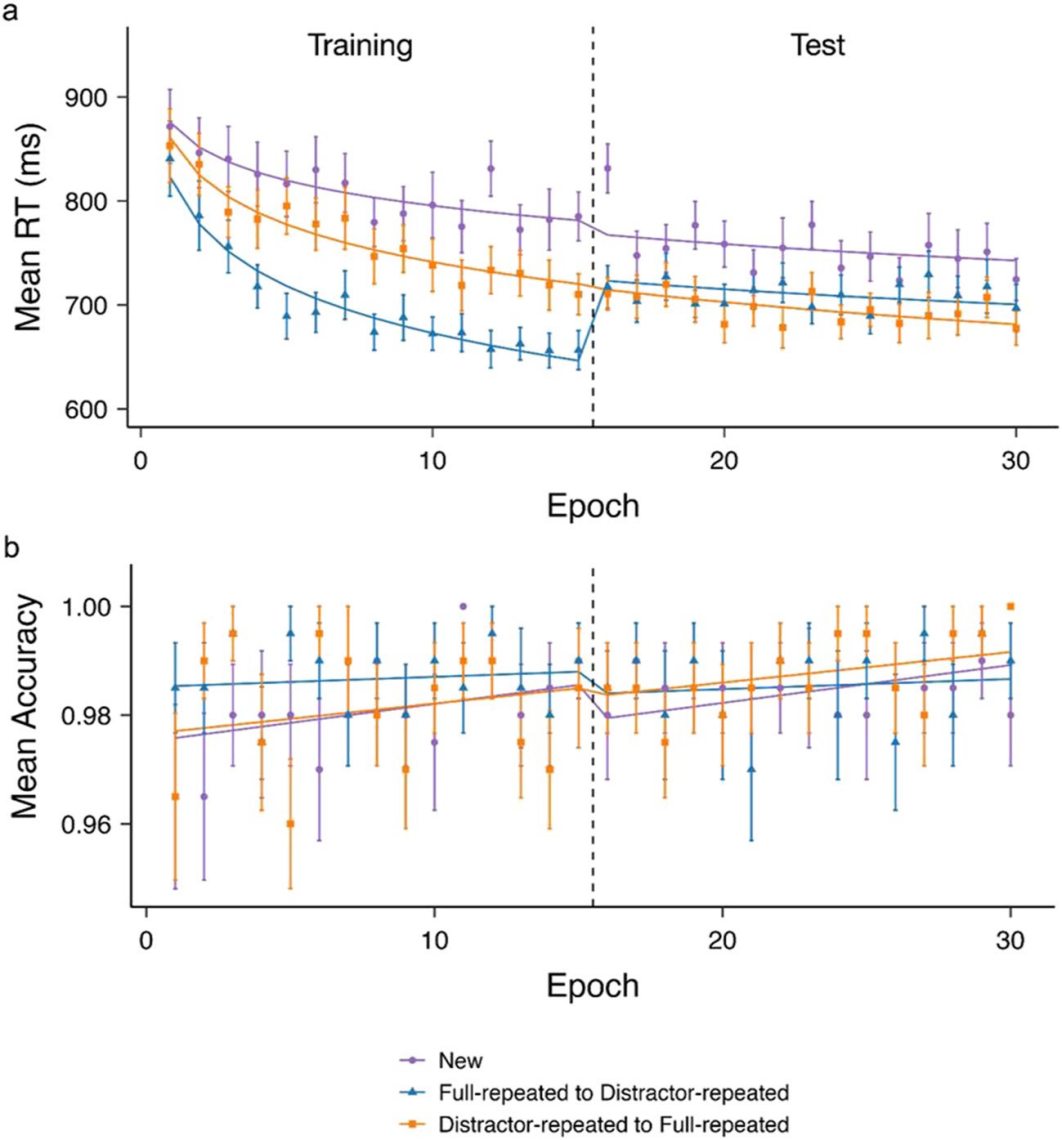
Mean reaction time (RT) (**a**) and mean accuracy (**b**) observed during the experiment (dots) and RT/accuracy estimated by the linear-mixed-model analyses (LMMs) (lines) for the various display types as a function of Epoch number (each epoch averaging two blocks), separately for training and test phases (with the transition marked by a dashed vertical line). The transition from “full-repeated to distractor-repeated” indicates that the old contexts in initially full-repeated displays (in the training phase) were rendered non-predictive of the target location (in the test phase), while the distractor configurations remained the same. The transition from “distractor-repeated to full-repeated” indicates that the old contexts in initially distractor-repeated displays (in the training phase) rendered fully predictive of the target location (in the test phase), without any change in the distractor configurations. Error bars indicate the within-subject standard error of the mean

Figure 2a presents the observed RTs (indicated by the dots with associated error bars) and predicted RTs (indicated by the curves) from the LMM. We used the novel displays as a baseline for comparisons. In terms of the LMM intercept (at Epoch 1), new displays showed a mean RT of 875.83 ms (*a* = 875.83 [835.23, 916.42], *p* < 0.001). Compared to new displays, full-repeated displays exhibited a significant gain in RT (*a* = -52.66 [-82, -23.32], *p* < 0.001), whereas distractor-repeated displays failed to show a significant decrease (*a* = -14.82 [-44.16, 14.53], *p* = 0.32). A repeated-measures ANOVA on the very first block of task performance revealed no significant effect of Display (*F* (1.88, 45.07) = 0.54, *p* = .58, *η*_*p*_^*2*^ = .02), suggesting the significant Epoch intercept effect derived mainly came from block 2 (of Epoch 1).

We used the absolute slope of log(Epoch) as the learning rate. The baseline learning rate with novel displays was 34.87 ms per log(Epoch) (*b* = -34.87 [-45.22, -24.51], *p* < 0.001). The learning rate was increased by 30.39 ms per log(Epoch) for full-repeated (relative to novel) displays (*b* = -30.39 [-45.03, -15.75],* p* < 0.001), and by 17.10 ms per log(Epoch) for distractor-repeated displays (*b* = -17.10 [-31.74, -2.45],* p* = 0.02). Relative to procedural learning in the (no-context) baseline condition, this pattern is indicative of additional, context-dependent learning with both types of display.

Crucially, there was interaction between log(Epoch), Display and Phase, marked by a significant decrease in the learning rate (decreasing 29.21 ms per log(Epoch)) when full-repeated displays in the training phase transformed into distractor-repeated displays in the test phase (*l* = 29.21 [23.67, 34.74],* p* < 0.001). Critically, the transition from distractor-repeated to full-repeated displays did not yield a significant phase difference (*l* = -0.84 [-6.38, 4.69],* p* = 0.77), nor did the change of the (within a given phase fixed) target locations in new displays (*l* = -4.26 [-9.80, 1.27],* p* = 0.13). In other words, only the shift from full-repeated to distractor-repeated displays engendered a transition cost.

#### Accuracy

Accuracies fluctuated between 96% and 100% (Fig. 2b), exhibiting no discernible trends. The mean accuracies were 98.6% for the full-repeated to distractor-repeated display-change condition, 98.4% for the distractor-repeated to full-repeated condition, and 98.3% for the new-display condition. The LMM failed to yield any effects, all *p*s >.05. The accuracies were also comparable in the very first block of task performance (*p* = .48, *η*_*p*_^*2*^ = .03).

#### Transition cost/benefit

To explore the phase-transition cost, we conducted a repeated-measures ANOVA with the within-subject factors Phase (training, test) and Display (full-repeated to distractor-repeated, distractor-repeated to full-repeated, and new displays) comparing the mean RTs between the last two epochs (Epochs 14 and 15) in the training phase with those in the first two epochs (Epochs 16 and 17) in the test phase (see Fig. 2a). This ANOVA yielded a significant main effect of Display (*F*(1.95, 46.72) = 35.23, *p* < .001, *η*_*p*_^*2*^ = .59) and a marginal effect of Phase (*F*(1, 24) = 3.59, *p* = .07, *η*_*p*_^*2*^ = .13), as well as a significant Phase × Display interaction (*F*(1.92, 46.17) = 4.06, *p* = .025, *η*_*p*_^*2*^ = .14). Specifically, there was a significant *increase* in RT when the full-repeated displays transformed into distractor-repeated displays (mean difference = -53.88 ms, *p* < .001, *d*_*z*_ = -0.79; one-tailed t-test was applied to all pairwise comparisons). However, there was no significant difference when the distractor-repeated displays changed into full-repeated displays (mean difference = 5.45 ms, *p* = .64, *d*_*z*_ = 0.07), or for the novel displays across the two phases (mean difference = -5.53 ms, *p* = .39, *d*_*z*_ = -0.06). Further, there was a significant difference between the (initially) full-repeated and (initially) distractor-repeated displays in the training phase (mean difference = -58.75 ms, *p* = 0.001, *d*_*z*_ = -0.68), but no difference between them after the transition (mean difference = 0.58 ms, *p* = 0.52, *d*_*z*_ = 0.008). In both the training and the test phases, both full-repeated and distractor-repeated displays engendered faster RTs than novel displays (training: full-repeated vs. new: mean difference = -126.20 ms, *p* < 0.001, *d*_*z*_ = -1.34; distractor-repeated vs. new: mean difference = -67.44 ms, *p* = 0.003, *d*_*z*_ = -0.62; test: full-repeated vs. new (post-transition): mean difference = -77.85 ms, *p* < 0.001, *d*_*z*_ = -1.14; distractor-repeated vs. new: mean difference = -78.43 ms, *p* < 0.001, *d*_*z*_ = -1.22).

An analogous ANOVA of performance accuracy in the last epoch in the training and the first epoch in the test phase (see Fig. 2a) revealed no significant effects (*all p*s > .47, *η*_*p*_^*2*^s < .02). The accuracies for the full-repeated, distractor-repeated, and new displays showed no discernible differences across the two phases (98.5%, 97.8%, and 98.8%, respectively, in the training phase, compared to 98.5%, 98.8%, and 98.5%, respectively, in the test phase).

#### Recognition task

Participants’ explicit recognition performance – that is, their ability to tell apart repeated displays (“signals”) from non-repeated displays (“noise”) – was assessed by the signal-detection sensitivity parameter *d’* (Green & Swets, 1966), treating correct recognition of repeated displays as “hits” and incorrect “recognition” of novel displays as “false alarms.” The mean *d’* score (*d’* = 0.25) turned out greater than zero, *t* (24) = 3.55, *p* = .002,* d* = 0.71 – making it possible that participants had learnt to explicitly recognize (at least some of the) repeated displays during the search task.

## Discussion

Using a training-/test-phase design, the present study investigated how acquired contextual-*guidance* and context-*suppression* mechanisms adapt to sudden but persistent changes in target-location predictivity across the two phases. During the training phase, participants encountered three types of display: full-repeated displays with fixed target-context arrangements; novel displays with randomly placed distractors; and distractor-repeated displays with fixed distractor contexts, but (across repetitions) randomly variable target locations. In the subsequent test phase, in addition to newly generated novel displays, the target location in (initially) full-repeated displays in the training phase was randomized (transforming them into distractor-repeated displays); conversely, the target location in (initially) distractor-repeated displays in the training phase was fixed (transforming them into full-repeated displays). As expected, a contextual-facilitation effect was observed for both full-repeated and distractor-repeated displays, evidenced by more pronounced RT gains over time-on-task (epochs) compared to novel displays. Crucially, there was an interaction between Display and Phase in the learning rate, owing to a significant decrease in the learning rate after full-repeated displays in the training phase transformed into distractor-repeated displays in the test phase. In contrast, there was no significant difference for the transition from distractor-repeated to full-repeated displays (and there was no phase difference for new displays). That is, only the shift from full-repeated to distractor-repeated displays engendered a transition cost. This was corroborated by direct comparisons of the last two epochs of training versus the first two epochs of the test phase, which revealed a significant RT cost when full-repeated displays transformed into distractor-repeated displays, but no cost, or gains, when distractor-repeated displays turned into full-repeated displays, and no cost associated with the change of the target locations in novel displays. In both the learning and the test phases, RTs were significantly faster compared to the novel-display baseline for both types of repeated displays, evidencing contextual-facilitation effects. However, while the facilitation effect was larger for full-repeated versus distractor-repeated displays in the last epochs of the training phase, it was no longer greater (numerically, it was smaller) after transition.

Overall, our results indicate that RTs decreased faster, across time-on-task (epochs), for both full-repeated and distractor-repeated displays compared to novel displays, during both the training and test phases, indicative of contextual learning. For novel displays, the learning rate remained stable even though a different set of target locations was introduced in the test phase. Since new displays afford no contextual learning, learning in new displays is considered to largely reflect general procedural task learning (cf. Seitz et al., 2023).^2^ The expedited learning rates in the two “repeated” conditions confirm that contextual learning can occur even in the absence of any fixed relationship between the target and the distractor configuration, as long as there is an invariant distractor configuration across blocks. These findings replicate both the classic contextual-cueing effect (Chun & Jiang, 1998) and Vadillo et al.’s (2021) observation that repeated contexts with random target locations alone can give rise to facilitated search performance, though the facilitation is less with such distractor-repeated than with full-repeated displays.

This pattern of results can be explained by assuming that, in the training phase, observers come to associate, and then operate, two different modes of search with the two types of repeated display: contextual-guidance with full-repeated displays and context-suppression with distractor-repeated displays. In full-repeated displays, the target location is uniquely predicted by the distractor configuration, so observers learn to use this configuration to direct attention to the target location. That is, activation of the distractor context enhances, via acquired associative links, the priority of the target location, making the target more competitive for summoning (overt or covert) attention (cf. Brady & Chun, 2007). In distractor-repeated displays, by contrast, the distractor context does not predict the target location. So, a relatively more efficient processing mode would be to (learn to) lower the priority of – that is, suppress – the (associatively linked) distractor locations. This context suppression would leave the target, which is not associatively linked with the distractors, unaffected: its priority signal would not be lowered. However, by reducing the signaling of the distractors, context suppression would confer an advantage to the target in the competition for attentional selection, wherever the target appears in the display. The target-directed contextual-guidance mode is more efficient than the context-suppression mode, conceivably because full-context learning raises the priority of the target location more compared to the activation difference between the target and the distractor locations when operating in distractor-context suppression mode. This would explain why, in the initial training phase, relative to new-display baseline, the contextual learning rate and learning gains are larger for full-repeated versus distractor-repeated displays (a pattern consistent with Vadillo et al., 2021).

In the test phase, the initially distractor-repeated displays are turned into full-repeated displays (i.e., the target location now becomes predictable), while the initially full-repeated displays are turned into distractor-repeated displays (i.e., the target location is now non-predictable). Of note, in both conditions, the (four) target locations are changed, that is, targets do not appear at any of the locations occupied by targets in the preceding training phase. Now, initially distractor-repeated displays still trigger the context-suppression mode in the test phase, which does curtail the learning of the newly fixed target locations: learnt suppression of the distractor configuration prevents the target location from being effectively linked to this configuration, limiting the acquisition of contextual guidance (see also Kunar & Wolfe, 2011). This would explain why, in the distractor-repeated to full-repeated transition condition, observers perform in exactly the same way in the test phase as in the training phase, even though the test phase offers the opportunity for contextual guidance: witness, in Fig. 2a, the “seamless” continuation of the RT curve across the transition, with the decreasing slope (across epochs) in the two phases showing no discernible difference.

In contrast, the transition from full-repeated to distractor-repeated displays gives rise to a qualitative shift in performance: the absolute RT gain from the preceding training phase is diminished and the learning rate is reduced in the test versus the training phase. This shift can be attributed to the sudden (and then continuing) failure of the contextual-guidance mode after the change in context predictivity: while the initially acquired contextual cues would still guide attention to the predicted target location, that location is no longer occupied by a target – causing a “mis-guidance” cost (cf. Zellin et al., 2013, 2014; Zinchenko et al., 2020). Interestingly, however, this cost is not as large as in previous “target-relocation” studies, where performance fell back to the baseline level and it took a massive amount of practice to re-integrate the new target location into the previously learnt distractor context (e.g., Zellin et al., 2013, 2014). In the present study, the general performance level is only insignificantly worse than in the reverse (i.e., distractor-repeated to full-repeated) transition condition, but significantly better compared to the novel-display baseline. This suggests that observers did not persist in operating a contextual-guidance mode, partly because this mode produced persistent misguidance and partly because the non-predictable positioning of the target location relative to the distractor-repeated context rendered attempts to integrate the new target locations in the old contexts futile. As a result, observers may have switched to a contextual-suppression mode, potentially aided by the fact that the distractor contexts themselves remained the same as in the training phase. That is, they (relatively flexibly) switched mode, thus capitalizing on what had been learnt previously, rather than engaging in any new learning. This would be similar to the classical notion of “latent inhibition,” accounting for the difficulty, in learning, to associate something new with an already familiar stimulus (Lubow, 1973; Lubow & Kaplan, 2005).

Thus, the assumption of two qualitatively different acquired context-processing modes, together with the constraints imposed by the context conditions on what information can be extracted and utilized after the switch, would coherently explain the present pattern of findings.

The above account appears most compatible with Beesley et al.’s (2015) (neuro-) computational model scheme, which was designed to account for their finding that pre-exposure to repeated distractor configurations subsequently enhances contextual cueing of (in relation to the configurations) consistently positioned target items. Their model assumes that learning of repeated display arrangements involves the acquisition of not just distractor-target associations – in the facilitatory links connecting “distractors” in the input layer/map to the target in the output (i.e., the search-guiding priority) layer/map (involving supervised learning), as in Brady and Chun’s (2007) model, but also of associations among the invariantly placed distractors – conceivably implemented by inhibitory links among distractors within the input map (involving unsupervised learning). That is, although the inhibitory (suppressive) and facilitatory mechanisms are separate, they would normally operate in tandem: with full-repeated displays, the system always operates in both the context-suppression and contextual-guidance modes, and the resulting contextual facilitation would be the additive effect of both mechanisms (consistent with Ogawa et al., 2007,3). In contrast, the scheme envisaged in our account involves a relatively flexible switching between separable modes, in particular: switching from contextual guidance to contextual suppression. In a model scheme along the lines of Beesley et al. (2015), this would be equivalent to re-initializing (or un-learning of) the weights connecting (distractors in) the input layer to (targets in) the output layer.

Of course, this is only one of various alternative “model” implementations (see, e.g., Seitz et al., 2024, for a “procedural” learning model that successfully simulates contextual facilitation, without “knowing” anything about which item is a target or a distractor). Of note, Beesley et al. (2015) actually conceives of the “auto-associative” links among the distractor items as facilitatory, strengthening the context-target association – contrasting with the notion of “latent inhibition” (see above). Yet another explanation for the reduced cueing effect and learning rate following the transition from full-repeated to distractor-repeated displays might be that the distractor configuration somehow loses its salience over time, owing to its diminished associability with a target location (cf. Mackintosh, 1975). Accordingly, further empirical and modeling work would be required to corroborate our “distinct-mode” assumption (vs. alternative assumptions of “mode-mixing”) and how this may be implemented in connectionist terms and in the brain.

## Data Availability

The data and materials have been made available via the Open Science Framework and can be accessed at: https://osf.io/pjq2f/.

## Footnotes

1 Conceivably, also, both may be learnt, producing additive effects. This would be consistent with Ogawa et al. (2007): using a dot-probe detection task, they found detection of the probe to be facilitated at the target location and inhibited at distractor locations in repeated relative to non-repeated target-distractor arrangements. We consider the possibility of additive effects further in the *Discussion*.

2 Of course, the four recurrent target locations may also be learnt in novel displays, so the improvement in performance may also involve some absolute target-position learning. However, since there were also four recurrent target locations in the full-repeated and distractor-repeated conditions, any absolute target-position learning would be equated across all display conditions and the expedited learning rates (relative to novel displays) in the latter two conditions are attributable to contextual learning.

3 Note that Ogawa et al.’s (2007) evidence, based on the dot-probe RTs, for context suppression in full-repeated displays is somewhat equivocal with regard to when, during search performance, the suppression actually arises: post-selectively, i.e., as a by-effect of target selection (cf. Müller et al., 2007), or pre-selectively, actually facilitating target selection.
